# A comparison of the physical activity levels of 3-to-6-year-old children with autism spectrum disorder and children with typical development

**DOI:** 10.3389/fpsyg.2024.1432389

**Published:** 2024-09-04

**Authors:** Jing Wang, Jiaxin Yao, Yingli He

**Affiliations:** ^1^School of Education and Psychology, Tianjin University of Sport, Tianjin, China; ^2^Key Laboratory of Competitive Sport Psychological and Psychological Regulation, Tianjin University of Sport, Tianjin, China; ^3^Tianjin Rehabilitation Service Guidance Center for the Disabled, Tianjin, China

**Keywords:** autism, child development, physical activity, children, exercise

## Abstract

**Background:**

Physical activity during early development is closely related to health. Differences in physical activity between young children with autism spectrum disorder and those with typical development are unclear. The purpose of this study was to compare the physical activity levels in children with autism spectrum disorder and typically developing children from the same area, including their sedentary physical activity, light physical activity, moderate-to-vigorous physical activity, and number of days in which the moderate-to-vigorous physical activity guideline recommendation of 60 min per day was met.

**Methods:**

A total of 77 participants aged 3–6 years were included: 41 children with autism spectrum disorder (mean age = 61.41 ± 10.69 months) and 36 children with typical development (mean age = 60.36 ± 10.16 months). The physical activity of the children was measured using an ActiGraph GT3x accelerometer.

**Results:**

There were no significant differences in daily sedentary physical activity (439.70 ± 54.98 vs. 450.42 ± 53.67) or moderate-to-vigorous physical activity (46.62 ± 18.93 vs. 47.47 ± 18.26) between the two groups. The average daily moderate-to-vigorous physical activity of the two groups did not reach 60 min, and they had similar proportions of participants who reached 60 min a given number of times (24.4% vs. 25%). Daily light physical activity was significantly higher in the autism spectrum disorder group (263.96 ± 43.17 vs. 242.32 ± 37.91, *p* < 0.05). The moderate-to-vigorous physical activity of both groups was similar and lower than the recommended minimum physical activity.

**Conclusion:**

Targeted interventions should be considered in early intervention programs for children with autism spectrum disorder to increase their moderate-to-vigorous physical activity.

## 1 Introduction

Autism spectrum disorder (ASD) is a pervasive neurodevelopmental disorder whose symptoms begin before 3 years of age and whose core features are social communication and interaction deficits, repetitive stereotyped behavior patterns, and narrow interests (American Psychiatric Association, 2013). Children with ASD often have motor skills impairments, including poor motor coordination, balance, and difficulty executing motor sequences (Minshew et al., 2004; Bhat et al., 2011; Kaur et al., 2018).

The World Health Organization (2020) recommended that children and adolescents perform at least 60 min of moderate-to-vigorous physical activity per day to obtain health benefits. The benefits of physical activity for typically developing children and adolescents are well documented. Physical activity also has positive effects on the physiology and psychology of children with ASD, including the ability to promote advanced motor skill development (Bremer and Lloyd, 2021), improve social skills (Zhao and Chen, 2018) and cognitive function (Memari et al., 2017), and reduce challenging behaviors (Anderson-Hanley et al., 2011).

Children with ASD often show low physical activity levels, with only 14% who reach the minimum recommendations of the WHO guidelines (Case et al., 2020). Some scholars have argued that the social and behavioral impairments of children with ASD may prevent them from participating in physical activities (Must et al., 2015; Yu et al., 2018). More specifically, impairments in social and communication skills may limit activities that require cooperation, such as team sports or group games (Golden and Getchell, 2017). For children with ASD, motor skill difficulties are a significant barrier to participation in physical activity and may limit their opportunities to successfully participate in physical activity (Srinivasan et al., 2014; Bull et al., 2020). Other studies have attributed the inactivity of children with ASD to social and environmental factors such as limited resources, staff training, family and community factors (Obrusnikova and Miccinello, 2012; Memari et al., 2015; Gregor et al., 2018; Brown et al., 2020). Limited physical activity level is associated with several negative health outcomes such as obesity and lower bone density (Kohl et al., 2012; Neumeyer et al., 2013; Broder-Fingert et al., 2014; Garcia-Pastor et al., 2019). Therefore, it was necessary to measure and understand the physical activity of children with ASD to determine whether relevant interventions should be introduced or modified.

Most studies on physical activity in children with ASD have focused on moderate-to-vigorous physical activity. In moderate-to-vigorous physical activity studies comparing children with ASD and typically developing children, some revealed differences between the two groups, while others did not. Age may be a factor determining whether a difference is observed (Pan and Frey, 2006; MacDonald et al., 2011; Memari et al., 2013; Jones et al., 2017). When examining older adolescents, the moderate-to-vigorous physical activity of children with ASD was significantly lower than that of typically developing children of the same age (Pan et al., 2011, 2015; McCoy et al., 2016), and was lower than the guideline recommendation of 60 min per day (Stanish et al., 2017; McCoy and Morgan, 2020). A recent meta-analysis also found that individuals with ASD spend 30 min less per day engaging in moderate-to-vigorous physical activity than their typically developing peers (Rostami Haji Abadi et al., 2023). This difference is often attributed to the fact that the features of ASD, such as high anxiety, difficulty with social interactions, and a preference for structured and repetitive activities, are more evident in older children (Borremans et al., 2010; Stanish et al., 2015). When the participants were younger, the children with ASD engaged in moderate-to-vigorous physical activity for a similar length of time as typically developing children (Sandt and Frey, 2005; Bandini et al., 2013; Thomas et al., 2019); thus, their daily moderate-to-vigorous physical activity met the recommended 60 min (Obrusnikova and Cavalier, 2011; Tyler et al., 2014; Ketcheson et al., 2018). However, children with ASD under 6 years of age may have different physical activity patterns (Memari et al., 2017), and previous studies have reported different findings. For example, Thomas et al. (2019) used an accelerometer to measure moderate-to-vigorous physical activity in children aged 4–7 years with ASD and those with typical development. Their results confirmed that the moderate-to-vigorous physical activity levels of the children in both groups reached similar levels of at least 60 min per day. Ketcheson et al.’s (2018) study showed that although the moderate-to-vigorous physical activity of both groups of children aged 2–5 years reached 60 min, the moderate-to-vigorous physical activity of children with ASD was significantly greater than that of typically developing children of the same age. On the contrary, Sung et al. (2021) found that children with ASD spent significantly less time in moderate-to-vigorous and light physical activity than did children with typical development.

The early physical activity of an individual is very important. The formation of early physical activity habits affects physical activity levels in adolescence and adulthood (Jones et al., 2013; Telama et al., 2014). Successful physical activity experiences in childhood may be highly correlated with health status in adulthood (Colombo-Dougovito et al., 2021). Moreover, young children are in the critical period of motor skills and ability development, and participation in physical activity may promote the development of these skills (Figueroa and An, 2017). To design high-quality and evidence-based physical activity interventions in the critical period development for children with ASD, it is necessary to understand the levels of physical activity. Most research on comparing physical activity levels in those with ASD and their neurotypical peers were based on school-age children and adolescents. Limited studies have compared the levels of physical activity in young children with and without ASD (Rech et al., 2022), and it is difficult to draw conclusions about these limited data and the conflicting findings. In addition, lower levels of time spent in sedentary behaviors being beneficial for health and replacing sedentary time with any intensity of physical activity (including light intensity) has health benefits even among those with modest levels of moderate-to-vigorous physical activity (Bull et al., 2020). Comparative studies of light physical activity are also necessary in view of the health benefits of light physical activity (Piercy et al., 2018; Wang et al., 2021) and the possibility that light physical activity will reveal differences in physical activity patterns between children with ASD and typically developing children.

Therefore, in the present study, children with ASD and typical development aged 3–6 years participated in a study that measured their daily physical activity by accelerometer-based objective measurements.

We aimed to (1) compare the physical activity levels of young ASD children and typically developing children in the same area, including their sedentary physical activity, light physical activity, and moderate-to-vigorous physical activity; and (2) determine the situations in which the daily moderate-to-vigorous physical activity of children with ASD met the minimum for recommended totals.

We hypothesize that the light and moderate-to-vigorous physical activity of young children with ASD is significantly lower than that of typically developing children of the same age.

## 2 Materials and methods

A cross-sectional design was used in this study.

### 2.1 Participants

We used G*Power 3.1 to calculate the sample size. A priori power analysis indicated that a total of 68 participants was necessary to reach the 90% statistical power level in the independent-samples *t*-test (*α* = 0.05, effect size = 0.8, two-tailed). Participants with ASD and typical development aged 3–6 years were recruited from the same geographical area (Tianjin) in China. Participants with ASD were recruited through the Tianjin Rehabilitation Center for the Disabled and Tianjin Rehabilitation Association for Children with ASD. Children with typical development were recruited through community-based organizations. All participants were good health and had no illnesses. The exclusion criteria for both groups included chronic illness, physical disability, injury, or any other factors limiting physical activity.

Children with ASD were required to have a formal diagnosis of ASD. A total of 95 children were included in the study. All participants provided informed consent from their parents and signed informed assent before participating in the study. Participants could withdraw from the study at any time without consequence. Due to factors such as children dropping out of the study for personal reasons and daily wearing time not meeting the requirements, the data of 18 patients were invalid and excluded from the analysis. The final sample comprised 77 participants with valid data, including 41 children with ASD and 36 children with typical development. All children with typical development and 13 children with ASD were enrolled in kindergartens, while 28 children with ASD were enrolled in kindergartens for children with ASD. Table 1 shows that the ASD group was slightly older than the typically developing group; however, the difference was not significant (61.41 months vs. 60.36 months, *p* > 0.05). The ASD group had a slightly higher proportion of boys.

**Table 1:** Descriptive data and physical activity level for both groups.

Variable	ASD (*n* = 41), *M* ± SD	TD (*n* = 36), *M* ± SD	*p* value
Age (months)	61.41 ± 10.69	60.36 ± 10.16	0.66
<60 months (%)	18 (43.90)	14 (38.89)	
≥60 months (%)	23 (56.10)	22 (61.11)	
Sex			
Male (%)	29 (70.7)	21 (58.3)	
Female (%)	12 (29.3)	15 (41.7)	
BMI (kg/m^2^)	15.56 ± 1.95	14.37 ± 2.28	0.02^*^
Normal weight (%)	26 (63.41)	28 (77.78)	
Overweight/obesity (%)	15 (36.59)	8 (22.22)	
Wear time (min/d)	750.28 ± 81.72	740.22 ± 74.30	0.57
SPA (min/d)	439.70 ± 78.35	450.42 ± 53.67	0.48
LPA (min/d)	263.96 ± 43.17	242.32 ± 37.91	0.02^*^
MPA (min/d)	34.99 ± 14.23	36.38 ± 12.85	0.65
VPA (min/d)	11.63 ± 6.53	11.09 ± 7.00	0.72
MVPA (min/d)	46.62 ± 18.93	47.47 ± 18.26	0.84

ASD, autism spectrum disorders; TD, typically developing; SPA, sedentary physical activity; LPA, light physical activity; MPA, moderate physical activity; VPA, vigorous physical activity; MVPA, moderate-to-vigorous physical activity. Mean ± SD (standard deviation), ^*^p < 0.05.

### 2.2 Instrument

#### 2.2.1 Demographics

Demographic data included age, gender, and body mass index (BMI). Parents completed a questionnaire with the age and gender of their children. The height and weight of the participants were measured with a portable weighing scale. Body mass index (BMI) was calculated according to the formula.


$$BMI=\mathrm{weight}\;\left(\mathrm{kg}\right)/\mathrm{height}\;{\left(m\right)}^{2}$$

We used the criteria of Li et al. (2010) to divide the children into normal-weight, overweight, and obese.

#### 2.2.2 Physical activity

Physical activity was measured using the ActiGraph GT3x accelerometer. It can be used to measure habitual physical activity accurately in young children (Cliff et al., 2009a), including those with ASD (Jones et al., 2017). ActiGraph GT3X is a triaxial tool measuring accelerations in three different planes: mediolateral, vertical, and anteroposterior planes. The children wore an accelerometer on their right iliac crest. The test lasted for 7 days, and the accelerometer was worn all day except while sleeping, swimming, and bathing.

Actigraph data are in counts per time intervals and represent the intensity of the activity during each period. Relevant studies have demonstrated the validity of an accelerometer sampling interval of 15 s for measuring physical activity in children (Cliff et al., 2009b; Colley et al., 2014). Therefore, in the present study, a sampling interval of 15 s was chosen to obtain the physical activity data. To distinguish between different intensity of physical activity, the Butte Preschoolers VM 2013 cutoff points was used. Cutoff points included sedentary physical activity (0–819 CPM), Light physical activity (820–3,907 CPM), moderate physical activity (3,908–6,111 CPM), vigorous physical activity (6112–) (Butte et al., 2014). Moderate-to-vigorous physical activity was calculated as the mean of the sum of moderate and vigorous physical activity.

Actilife software (Version 6.11.5) was used to download the data. The parameters for valid data were as follows: the daily wearing time must be ≥480 min; the non-wear time was defined as when the accelerometer count was 0 for 60 consecutive minutes; the Choi et al. (2011) algorithm was used to classify the non-wear time; and the wearing time was more than 4 days, including three weekdays and 1 weekend day (Hinkley et al., 2012).

### 2.3 Procedure

The children’s health and ASD diagnoses were determined through initial screening interviews, which were conducted to check their eligibility to participate in the study. The parents of those who were eligible to participate in the study gave their written informed consent allowing their children to participate in the study. The parents completed a questionnaire with basic information. Before test began, the research team explained the test contents and requirements to the parents in detail and instructed them on how to wear and remove the accelerometer. Study materials including the accelerometer, study instructions in written, and social-story video for wearing accelerometer were distributed to the parents. The parents were informed that they would receive a reward after returning the device. Participants completed an objective measurement of physical activity in the home environment for one week. The parents returned the equipment at the end of data collection.

The study protocol and all materials used were approved by the Ethics Committee of Tianjin Institute of Sport for the protection of human subjects.

### 2.4 Data analysis

The duration of each physical activity was expressed in minutes per day. First, a frequency analysis was performed on the participants’ gender, BMI, and whether daily moderate-to-vigorous physical activity met the minimum standard of 60 min recommended by the guidelines and the number of days on which this standard was met. The arithmetic means and standard deviation of the participants’ age, wearing time, sedentary physical activity, light physical activity, and moderate-to-vigorous physical activity were calculated. These data are presented as mean ± standard deviation. The independent-samples *t*-test was used to compare the physical activity of the two groups of children and examine the differences in the physical activity of children with ASD at different ages (<60 months, ≥60 months). The chi-square test was used to identify any significant difference between the two groups of children in meeting the standards recommended by the guidelines. The alpha level for statistical significance was set at 0.05, and all analyses were performed with SPSS 24.0.

## 3 Results

We obtained valid accelerometer data from 77 children. The average daily wearing times of the children in the ASD and typically developing groups were similar (*p* > 0.05), at 750.28 ± 81.72 min and 740.22 ± 74.30 min, respectively. The ASD group had a higher BMI than the typically developing group (15.56 kg/m^2^ vs. 14.37 kg/m^2^, *p* < 0.05).

The average number of times that ASD children engaged in sedentary physical activity and moderate-to-vigorous physical activity was slightly but not significantly lower than that of typically developing children (sedentary physical activity: 439.70 ± 54.98 vs. 450.42 ± 53.67, *p* > 0.05; moderate-to-vigorous physical activity: 46.62 ± 18.93 vs. 47.47 ± 18.26, *p* > 0.05). Their moderate physical activity and vigorous physical activity were also similar. There were no significant differences in sedentary physical activity, light physical activity, or moderate-to-vigorous physical activity between the two groups of children in any BMI stratum (normal, overweight/obese) or at any given age (<60 months, ≥60 months). The *t*-test showed that there were no significant differences in sedentary physical activity, light physical activity, or moderate-to-vigorous physical activity between ASD children in the two age groups we set. Light physical activity was significantly higher in the ASD group than in the typically developing group (263.96 ± 43.17 vs. 242.32 ± 37.91, *p* < 0.05) (Table 1).

On average, the moderate-to-vigorous physical activity of both ASD and typically developing children did not achieve the minimum 60 min/day recommended by the physical activity guidelines. The chi-square test showed no significant difference in the proportion of children who met the guidelines for moderate-to-vigorous physical activity between the two groups (24.4% vs. 25%, *p* > 0.05). Further statistical tests on the proportion of each group who met the recommendation different numbers of times showed that only one ASD child’s moderate-to-vigorous physical activity met the recommendation for all 7 days, and more than 60% of ASD and typically developing children reached 60 min zero or one time (Table 2).

**Table 2:** Met 60 min moderate-to-vigorous physical activity.

MVPA	ASD (*n*, %)	TD (*n*, %)	*p*
≥60 min/d	10 (24.4)	9 (25.0)	0.951
<60 min/d	31 (75.6)	27 (75.0)	
Days of met 60 min			
0 of days	15 (36.6)	15 (41.7)	
1 of days	11 (26.8)	9 (25.0)	
2 of days	3 (7.3)	5 (13.9)	
3 of days	3 (7.3)	1 (2.8)	
4 of days	2 (4.9)	1 (2.8)	
5 of days	5 (12.2)	3 (8.3)	
6 of days	1 (2.4)	2 (5.6)	
7 of days	1 (2.4)	0 (0)	

MVPA, moderate-to-vigorous physical activity.

## 4 Discussion

Young childhood is an important stage for the acquisition and solidification of individual life behaviors, and health behavior is more plastic. Based on the current studies, little is known about the differences in physical activity levels between young children with ASD and typically developing children of the same age. By exploring and comparing the physical activity levels of 3-to 6-year-old children with ASD and typically developing children, this study expanded upon the results of previous studies and provided more evidence for the difference between the physical activity levels of young ASD children and typically developing children of the same age.

On average, children with ASD and typically developing children had the same low moderate-to-vigorous physical activity levels; however, the difference between the two groups was not reach significant. This was in line with the results of several previous studies (Healy and Garcia, 2019; Thomas et al., 2019; Haegele et al., 2021). These results indicated that low moderate-to-vigorous physical activity levels in young children are less affected by the behavioral characteristics and social function of children with ASD, meaning that social or environmental factors, such as activity arrangements in kindergarten, may play a greater role. Moreover, family and community barriers impacted the moderate-to-vigorous physical activity levels of all young children. Ketcheson et al. (2018) compared the physical activity of 2-to 5-year-old children with ASD and typically developing children and found that children with ASD had more moderate-to-vigorous physical activity than typically developing children. These inconsistent results may be related to the small sample size, the younger mean age in that study (47 months), and the geographic differences between the samples. Therefore, further research is needed to determine whether there is a difference in moderate-to-vigorous physical activity between the two age groups.

Children with disability should try to meet physical activity recommendations where possible and as able (World Health Organization, 2020). Most studies find that children with ASD do not achieve moderate-to-vigorous physical activity of 60 min per day (Tyler et al., 2014; McCoy et al., 2016). Our study was no exception: the ASD group spent 46.62 min on moderate-to-vigorous physical activity per day. Only 24.4% of children with ASD met the minimum for recommended totals, close to the proportion (23%) reported by Bandini et al. (2013) in children with ASD, and lower than the proportions reported in similar studies (Sandt and Frey, 2005; Pan and Frey, 2006; Pan et al., 2015). In our study, 63.4% of the children with ASD met the minimum recommendation of 1 day per week. These findings indicated that young ASD children engage in too little moderate-to-vigorous physical activity and that improving moderate-to-vigorous physical activity is urgently necessary.

The level of physical activity changes throughout a child’s developmental process. As children get older and the game rules and motor skills required for play become more complex, children with ASD may not be able to adapt to competitive group games with their typical development peers (Arnell et al., 2018). The results of a study that objectively measured the physical activity patterns of adolescents with ASD aged 10–19 years showed that younger children spent significantly more time on moderate-to-vigorous physical activity than older children (Pan and Frey, 2006). The results of the present study were not consistent with these findings. Longitudinal analysis of the moderate-to-vigorous physical activity of children with ASD in the two age groups (<60 months, ≥60 months) revealed no significant differences, and moderate-to-vigorous physical activity was not affected by age in either group. The age range of the participants in this study was 36–78 months, which may indicate that early childhood was not a sensitive period for the start of changes in the moderate-to-vigorous physical activity trajectory. To explore age-associated factors, future studies should consider the longitudinal examination of the entire developmental trajectories of physical activity in ASD children and the age at which the group differences between ASD children and typical development children appear.

This study also compared light physical activity between typically developing and ASD children. The children with ASD engaged in significantly more light physical activity than did children with typically developing, at an average of 21 min more per day. This result is consistent with Ketcheson et al.’s (2018) study, which showed the proportion of daily light physical activity in children with ASD was significantly greater than that in typical development children. Haegele et al. (2021) compared age-, gender-, and BMI-matched adolescents with ASD and those with typical development and reported that the light physical activity of children was 30 min greater than that of typical development children. These large differences may be related to the special behavioral patterns of children with ASD (e.g., stereotyped behaviors such as body shaking). More comprehensive and refined measurements of how children with ASD accumulate their physical activity are needed. For example, studies should observe special behaviors such as stereotyped behaviors to determine whether the accumulation of physical activity in children with ASD is associated with such hallmark characteristics of ASD.

### 4.1 Limitations

Although the results of this study provide more evidence about physical activity in children with ASD and typical development in the early stage of development, this study has some limitations. First, the small sample size and higher proportion of boys with ASD may not reflect the physical activity of all young ASD and typically developing populations, limiting the interpretability of the results. Future studies should include more matched samples to examine the differences between the two groups. Second, while the use of an accelerometer may be a feasible way to assess physical activity intensity and frequency (Hinckson and Curtis, 2013), there is little empirical evidence validating the various cutoff points for physical activity in children with ASD, so the cutoff points in the present study may not be the best one. More studies are needed to better cutoff points for assessing physical activity in children with ASD. Finally, the accelerometers used in the present study could not detect water-based activities. Both accelerometers and questionnaires should be used as access methods in subsequent studies.

## 5 Conclusion

In general, moderate-to-vigorous physical activity was too low for both ASD and typical development children and did not meet the minimum guideline recommendations. Both groups had similar levels of moderate-to-vigorous physical activity. Therefore, the addition of moderate-to-vigorous physical activity to early intervention programs for children with ASD should be considered to better promote their health and their development. The children with ASD accumulated significantly more light physical activity than typical development children, a difference that future studies should consider in more depth.

## Data Availability

The raw data supporting the conclusions of this article will be made available by the authors, without undue reservation.
